# Supplementary material for: Photonic comb-rooted synthesis of ultra-stable terahertz frequencies
Source: Nat Commun. 2023 Feb 11;14:790. doi: 10.1038/s41467-023-36507-y (PMC9922295; doi:10.1038/s41467-023-36507-y)
Supplement: Supplementary file 1 — Supplementary Information [file 41467_2023_36507_MOESM1_ESM.pdf]

Supplementary Information for

Photonic comb-rooted synthesis of ultra-stable terahertz frequencies

Dong-Chel Shin, Byung Soo Kim, Heesuk Jang, Young-Jin Kim*, and Seung-Woo Kim*

Department of Mechanical Engineering, Korea Advanced Institute of Science and Technology (KAIST), 291 Daehak-ro, Yuseong-gu, Daejeon 34141, Republic of Korea.

*Corresponding authors: *swk@kaist.ac.kr, yj.kim@kaist.ac.kr*

1. Frequency noise power spectral density (PNPSD) of the proposed terahertz synthesizer.

The phase noise spectra given in Fig. 2c of the main text are converted to their corresponding frequency noise power spectral densities (FNPSDs) as presented in Figure S1. The FNPSD plots provide additional information for the performance comparison between our terahertz synthesizer and other state-of-the-art counterparts.

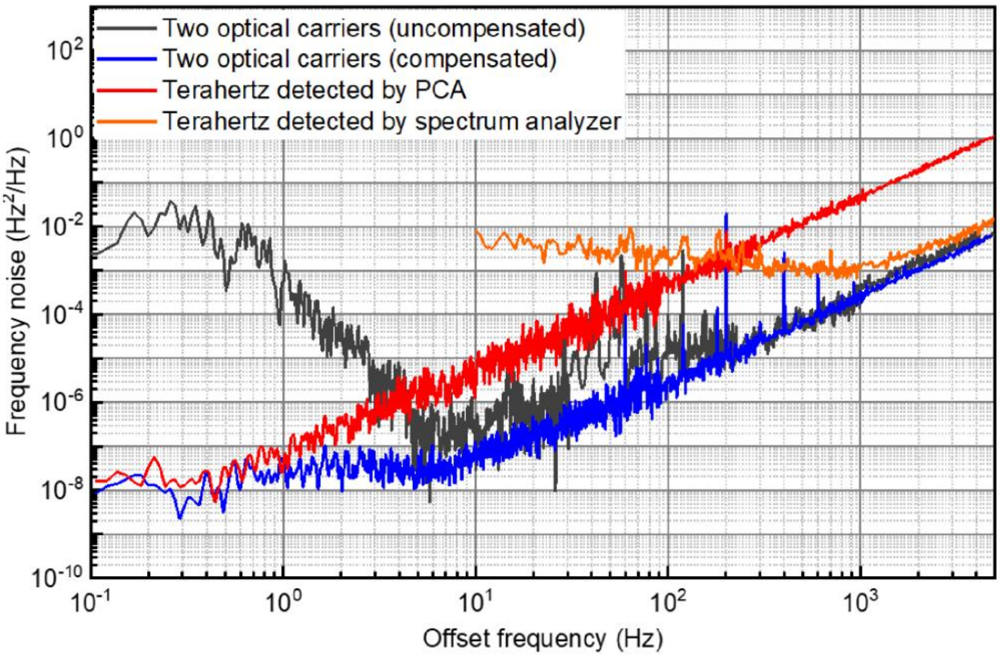

Figure S1 Frequency noise power spectral density (FNPSD) plots converted from the phase noise spectra given in Fig. 2c of the main text.

2. Spectral linewidth comparison.

Figure S2 presents the spectral linewidths of the terahertz waves measured with different stabilization conditions: (a) two free-running DFB lasers with no referencing to the source comb, (b) two comb lines before compensation of the fibre thermal noise, and (c) the same two comb lines after the thermal noise compensation. First, in the case of (a), the corresponding power spectrum shows no peak due to a large amount of thermal drift in the time domain as the DFB lasers are in a free-running state. Next, the case of (b) shows a coherent power spectrum having a spectral linewidth

of 1.46 Hz (FWHM) with a Lorentzian fit. Finally, in case (c), with the suppression of the fibre thermal noise as described in the main text, the spectral linewidth shrinks to 2 mHz, which in fact reaches the resolution limit imposed by the Fourier-transform made in this measurement with a sample period of 500 s. Such drastic linewidth improvement is found to hold true for all the terahertz frequencies of 0.10, 0.66, and 1.06 THz tested in this investigation as presented in Figure S3.

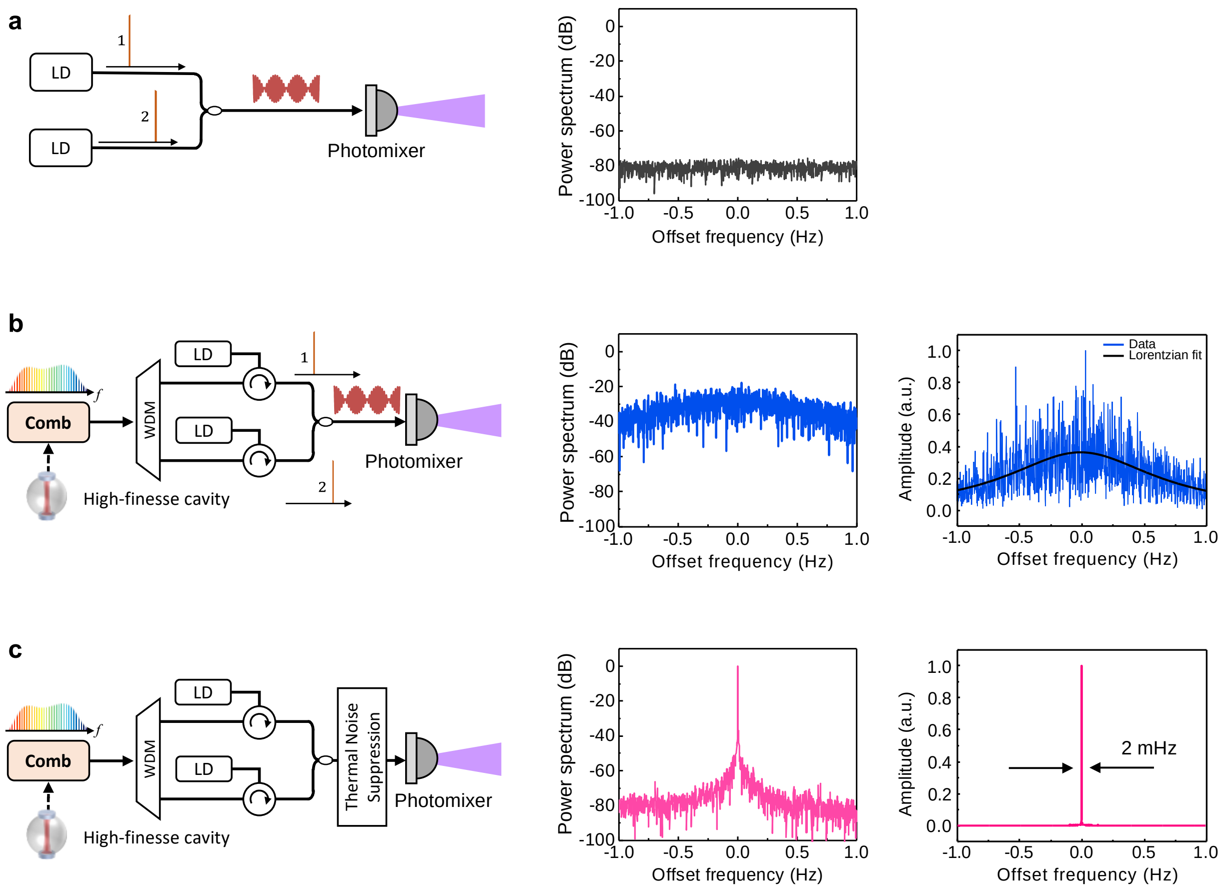

Figure S2. Comparison of the power spectra and spectral linewidths of the terahertz waves produced with three different conditions. a, Photomixing of two free-running distributed-feedback (DFB) lasers without comb-rooted injection locking. **b,** Photomixing of two comb lines before the suppression of the fibre thermal noise. **c,** Photomixing of the same two comb lines with thermal noise suppression. Note that the power spectrum of each case given on the right-hand side, in terms of the dB- and linear scale, is the result of the fast Fourier transform of a data set of 500-s sampling.

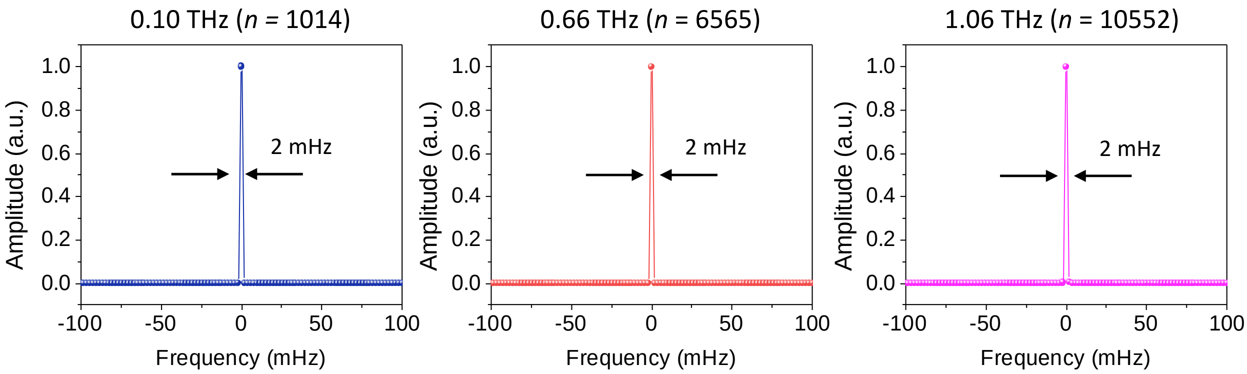

Figure S3 Spectral linewidths of terahertz waves. The terahertz frequencies of 0.10, 0.66, and 1.06 THz are determined with a resolution and a linewidth of 2 mHz, which is limited by the FFT limit.

3. Fractional frequency instability comparison

The fractional frequency instabilities marked with asterisks in Fig. 5a of the main text were calculated by taking the ratio of the given spectral linewidth to the given carrier frequency. For more details, the measurement parameters taken in our calculation are summarized below in Table S1.

Table S1. Calculation of fractional frequency instability

Ref. Number	Emitter type	Locking method	Frequency reference	Carrier frequency	Linewidth (definition)	RBW	Meas. time	Estimated fractional frequency instability
Barbieri, S. *et al.* (ref. 21)	QCL	PLL to comb	Free-running comb	2.7 THz	1 kHz (N/A)	N/A	N/A	3.7×10^{-10}
Ravaro, M. *et al.* (ref. 22)	QCL	PLL to comb	Free-running comb	2.5 THz	1 kHz (N/A)	N/A	N/A	4.0×10^{-10}
Freeman, J. R. *et al.*(ref. 23)	QCL	IL to terahertz waves	Microwave reference	2.0 THz	100 Hz (N/A)	N/A	N/A	5.0×10^{-11}
Quraishi, Q. *et al.* (ref. 31)	Photomixer	2 CW lasers/PLL to comb	Quartz crystal oscillator	0.3 THz	2 Hz (FWHM)	1 Hz	1 s	6.7×10^{-12}
Steed, R. J. *et al.* (ref. 33)	Photomixer	2 CW lasers/PLL to comb	Microwave reference	0.3 THz	1 kHz (FWHM)	N/A	N/A	3.3×10^{-9}
Criado, Á. R. *et al.* (ref. 35)	Photomixer	2 CW lasers/IL to comb	Microwave reference	0.14 THz	10 Hz (FWHM)	10 Hz	N/A	8.3×10^{-11}

4. SSB phase noise comparison

In Fig. 5b of the main text, the SSB phase noise values taken from references of 23, 31, 36, 37, and 38 were calibrated for the sake of fair comparison in two steps; First, each referenced SSB phase noise data set was quantified at the five distinct offsets in sequence at 1 Hz, 10 Hz, …, and 10 kHz. Then, the data set was scaled with respect to a nominal carrier frequency of 0.1 THz by incorporating the scale factor of –20 log (f_c/0.1THz) with f_c being the carrier frequency of each data set. The resulting raw numerical data adopted for comparison are shown in Table S2.

Table S2. SSB phase noise data for Fig.5b. Data shows scaled data (raw data). Unit: dBc/Hz

**Reference**	**Carrier frequency**	**Offset frequency**				
		**1 Hz**	**10 Hz**	**100 Hz**	**1 kHz**	**10 kHz**
(i) This work	0.1 THz	–71	–73	–73	–73	–74
(ii) Freeman, J. R. *et al.* (ref. 23)	2.0 THz	N/A	N/A	–96 (–70)	–98 (–72)	–98 (–72)
(iii) Quraishi, Q. *et al.*(ref. 31)	0.3 THz	N/A	–36.5 (–27)	–49.5 (–40)	–56.5 (–47)	–63.5 (–54)
(iv) Zhang, S. *et al.* (ref. 36)	0.331 THz	–40.4 (–30)	–49.4 (–39)	–47.9 (–37.5)	–59.4 (–49)	–83.4 (–73)
(v) Tetsumoto, T. *et al.* (ref. 37)	0.3 THz	N/A	N/A	–44.5 (–35)	–69.5 (–60)	– 94.5 (–85)
(vi) Tetsumoto, T. *et al.* (ref. 38)	0.3 THz	N/A	N/A	–39.5 (–30)	–67.5 (–58)	–119.5 (–110)
